# Elucidation of the Role of 3-Hydroxy Fatty Acids in *Cryptococcus*-amoeba Interactions

**DOI:** 10.3389/fmicb.2017.00765

**Published:** 2017-04-26

**Authors:** Uju L. Madu, Adepemi O. Ogundeji, Carolina H. Pohl, Jacobus Albertyn, Olihile M. Sebolai

**Affiliations:** Pathogenic Yeast Research Group, Department of Microbial, Biochemical and Food Biotechnology, University of the Free StateBloemfontein, South Africa

**Keywords:** 3-hydroxy fatty acids (3-hydroxy C9:0), amoeba, *Cryptococcus*, phagocytosis, shield

## Abstract

We previously reported that 3-hydroxy fatty acids promoted the survival of cryptococcal cells when acted upon by amoebae. To expand on this, the current study sought to explain how these molecules may protect cells. Our data suggest that 3-hydroxy fatty acids may subvert the internalization of cryptococcal cells via suppression of the levels of a fetuin A-like amoebal protein, which may be important for enhancing phagocytosis. Additionally, we show that an acapsular strain (that is devoid of 3-hydroxy fatty acids) was protected against the effects of hydrogen peroxide when exogenous 3-hydroxy fatty acids were present, but not in the absence of 3-hydroxy fatty acids. A similar response profile was noted when a strain with a capsule was challenged with hydrogen peroxide. We also show that cryptococcal cells that naturally produce 3-hydroxy fatty acids were more resistant to the effects of amoebapore (an amoeba-specific hydrolytic enzyme), compared to cells that do not produce these molecules. Taken together, our findings suggest that 3-hydroxy fatty acids possess an anti-phagocytic activity that may be expressed when cells interact with macrophages. This may allow the yeast cells to evade immuno-processing.

## Introduction

3-Hydroxy fatty acids are lipid-based molecules that are oxygenated and whose amphiphilic quality is evident when immersed in water (Tsitsigiannis and Keller, 2007; Sebolai et al., 2012). The chemical structure of these molecules is characterized by a hydroxyl group on the beta carbon from the carboxylic group – and their hydrocarbon chain can be linear or branched, saturated or unsaturated and may be linked to other macromolecules (Kock et al., 2003). It has been reported that these molecules are produced via an incomplete enzymatic pathway similar to mitochondrial beta oxidation (Kock et al., 2007; Sebolai et al., 2012). Herein, 3-hydroxy fatty acids are intermediate products that are poorly catalyzed by 3-hydroxyacyl-CoA dehydrogenase and consequently are secreted (Sebolai et al., 2008). This escape from the mitochondria suggests these molecules have no apparent function in the primary catabolism of the concerned organism.

It is thus not surprising that 3-hydroxy fatty acids are regarded as secondary metabolites that have, for example, been successfully implicated in microbial pathogenesis (Sebolai et al., 2012). To illustrate this point, Ciccoli et al. (2005) reported that *Candida albicans* can scavenge arachidonic acid from a host’s infected cells and convert it into a 3-hydroxy fatty acid (3-hydroxy eicosatetraenoic acid) via incomplete beta oxidation. In turn, the produced 3-hydroxy fatty acid can act as a substrate for the host’s cyclooxygenase-2 enzyme, leading to the production of 3-hydroxy prostaglandins, which are more potent pro-inflammatory mediators compared to non-hydroxylated prostaglandins.

The presence of 3-hydroxy fatty acids has also been documented in *Cryptococcus neoformans* (Sebolai et al., 2007). In an attempt to elucidate the function(s) of these molecules, Madu et al. (2015) studied how they may influence *Cryptococcus*-amoeba interactions. These researchers concluded that, at a physiological concentration, 3-hydroxy fatty acids protected cryptococcal cells by impairing the ability of amoeba to internalize and phagocytose cells. However, the mechanisms underlying this protective ability to 3-hydroxy fatty acids have not been elucidated yet. Therefore, the current study aims to determine how these molecules may impair the phagocytic process and/or protect internalized cells. This study may offer insight into how these molecules could assist cells to evade immuno-processing when acted upon by macrophages, which are said to have evolved from free-living amoebae (Siddiqui and Khan, 2012).

## Materials and Methods

### Strains, Cultivation, and Standardization

The fungal strains, *C. gattii* R265 (does not produce 3-hydroxy C9:0), *C. neoformans* LMPE 046 (does not produce 3-hydroxy C9:0), *C. neoformans* UOFS Y-1378 (produces 3-hydroxy C9:0), and *C. neoformans* LMPE 101 (acapsular strain that does not produce 3-hydroxy C9:0), were used in the study. These strains were grown on yeast-malt-extract (YM) agar (3 g/l yeast extract, 3 g/l malt extract, 5 g/l peptone, 10 g/l glucose, 16 g/l agar; Merck, South Africa) plates at 30°C for 48 h. Cells (representing the respective strains) were separately standardized using a haemocytometer (Marienfield, Germany) to a final concentration of 1 × 10^6^ cells/ml in 10 ml of distilled water before use. For amoebapore (BioWorld, United States) sensitivity testing, five colonies (representing either R265, LMPE 046, UOFS Y-1378, or LMPE 101) were scraped off and suspended in 10 ml of distilled water. At the end, the cells were standardized to prepare final inocula of between 0.5 × 10^5^ and 2.5 × 10^5^ CFU/ml in RPMI 1640 medium (Sigma–Aldrich, South Africa) according to EUCAST guidelines (Arendrup et al., 2015).

The amoeba (*Acanthamoeba castellanii*) strain LMPE 187, used in the study, was grown on peptone-yeast extract glucose broth, PYG (ATCC medium 30234^TM^) at 30°C for a week. The cells (in trophozoite state) were then standardized using a haemocytometer to a final concentration of 1 × 10^5^ cells/ml in 10 ml of sterile PYG broth and the viability of cells was determined to be 80% using trypan blue.

### 3-Hydroxy Fatty Acids

The 3-hydroxy fatty acid standard (3-hydroxy C9:0), was purchased from Larodan (Sweden). This compound was tested at a final concentration of 0.2 mM, which is the estimated physiological concentration secreted by *C. neoformans* UOFS Y-1378 (Madu et al., 2015).

### Glucuronoxylomannan (GXM) Isolation

Crude GXM was isolated in anticipation of experiments wherein it was used for comparison purposes. The isolation was done according to a protocol previously detailed by Zaragoza et al. (2008). In short, a loopful of scraped *C. neoformans* UOFS Y-1378 (grown for 48 h on YM plates) colonies was used to inoculate a 500 ml conical flask containing 250 ml of Difco-yeast nitrogen base (YNB) broth (6.7 g/l YNB and 40 g/l glucose; Becton, Dickinson and Company, United States) that was supplemented with 2% glucose (Merck, South Africa). The flask was placed on a rotary shaker (160 rpm/min) and cultivated for 48 h at 30°C. The cells were washed five times with distilled water and finally suspended in 40 ml of distilled water. The cells were then heat-killed at 55°C for 30 min before being irradiated (dosage of ∼200 grays) with a ^137^Cs gamma-irradiator (kept at HEPRO Cape, South Africa) in order to make cells shed their capsule (GXM). After irradiation, the cells were centrifuged at 3500 × *g* to mobilize the shed GXM into the supernatant.

Next, the lipids were removed from the collected supernatant via using a modified Folch lipid extraction protocol. In brief, the supernatant (10 ml) was transferred to a 50 ml Falcon tube (Becton-Dickinson Labware, United States). Following which, 10 ml of methanol-chloroform (HPLC-grade) solution (Merck, South Africa; 1:1, v/v) was added. The suspension was vortex mixed and allowed to stand for 20 min. Thereafter, distilled water (10 ml) was added to the above solution and allowed to stand for a further 20 min. The chloroform fraction that contained 3-hydroxy fatty acids was disposed and the water fraction containing the GXM was kept. The isolated GXM was then lyophilised, weighed and reconstituted in sterile water to make a stock solution of 10 mg/ml.

To confirm the isolation, 100 μl of the delipidated stock solution was subjected to an agglutination (Meridian Bioscience, Inc., United States) assay and ELISA (IMMY, United States) assay. Both assays were performed according to manufacturer’s protocol. The optical density (OD) readings were measured at 450 nm using a spectrophotometer (Biochrom EZ Read 800 Research, United Kingdom).

### Fetuin A and NOX-1 (NADPH Oxidase-1) ELISA Assays

A 100 μl suspension (in YPG broth) of standardized amoebae was seeded into designated wells of a sterile microtitre plate (Greiner Bio-One, Germany). Next, the cells were challenged with 3-hydroxy C9:0 (1:1, v/v) at a final concentration of 0.2 mM or 100 μl of isolated GXM to yield a final concentration of 2.5 mg/ml. Non-treated amoeba cells were included as a control. The plate was then incubated at 30°C for a 6 h period. At the end, the supernatant was aspirated and accordingly transferred to a sterile microtitre plate specific for the human-based fetuin A (Abcam, United Kingdom) while the cells were kept to harvest the lysate according to a protocol and materials provided in the NADPH oxidase-1 (human-based) kit (LifeSpan BioSciences, United States). The lysate was then transferred to a sterile microtitre plate specific for NADPH oxidase-1. The supernatant and lysate plates were, respectively, treated according to the manufacturer’s protocols. Each ELISA plate was then read at 450 nm using a Biochrom EZ spectrophotometer.

### The Effect of Hydrogen Peroxide on Cryptococcal Cells

Standardised cells (1 × 10^6^ cells/ml), in distilled water, of the strain LMPE 101 were used in this test. A 75 μl suspension of cells was separately transferred to wells of a sterile microtitre plate. These cells were then treated with 75 μl of hydrogen peroxide (60 μM). In other wells, cells (75 μl) were treated with 75 μl of hydrogen peroxide (240 μM) in the presence of either GXM (150 μl of 10 mg/ml) or 3-hydroxy C9:0 (150 μl of 0.8 μM). The plate was incubated for 3 h at 37°C. After incubation, the plate was gently agitated and the contents aspirated. Of this, 100 μl was used to make a 1:10 dilution in distilled water. Thereafter, 100 μl was used to create a uniform lawn of cells on a YM agar plate. The plates were incubated for 48 h at 30°C before colony forming units (CFU) were counted. The considered concentration of hydrogen peroxide was similar to that reported to accumulate inside phagosomes (Dupré-Crochet et al., 2013).

In addition, the levels of hydrogen peroxide in the medium containing hydrogen peroxide-treated cells and hydrogen peroxide-treated in the presence of GXM or 3-hydroxy C9:0 was measured as previously detailed by Noble and Gibson (1970). In brief, a microtitre plate was prepared as above-mentioned and at a specific time interval, i.e., 3-, 6-, and 24-h, an absorbance reading was taken at 360 nm. This reading was then multiplied by a molar extinction coefficient value of 43.6, in order to estimate the levels of hydrogen peroxide.

### The Effect of Amoebapore on Cryptococcal Cells

The effect of amoebapore (amoebal antimicrobial peptide) was independently assessed on biological duplicates of the strains R265, LMPE 046, UOFS Y-1378, and LMPE 101 at final concentrations (prepared in RPMI 1640 medium) of 3.25 μM or 7.5 μM. In short, a 100 μl of the standardized inoculum (0.5 × 10^5^ and 2.5 × 10^5^ CFU/ml) was aliquoted into designated wells of a microtitre plate. The cells, in the wells, were then treated with 100 μl of amoebapore (at twice the stated desired final concentrations). The plate was incubated for 48 h at 37°C. After 48 h, the OD of each well was measured at 562 nm using a Biochrom EZ spectrophotometer. The resultant effect of amoebapore on the growth of treated cells was compared to that of non-treated cells.

In order to assess if amoebapore killed cells by creating pores on cell walls, cells (i.e., UOFS Y-1378 and LMPE 101) treated with 7.5 μM, were prepared on a separate microtitre plate as stated above (for drug sensitivity testing) before being viewed by transmission electron microscopy (TEM). The cells were prepared for TEM viewing according to the method of van Wyk and Wingfield (1991). In short, the cells were chemically fixed with 1.0 M (pH 7) sodium phosphate-buffered gluteraldehyde (3%) for 3 h and then for 1.5 h in similarly buffered osmium tetroxide. The cells were next dehydrated in a graded acetone series, embedded in epoxy resin and polymerized at 70°C for 8 h. An LKB III Ultratome was used to cut 60-nm sections with glass knives. Uranyl acetate was used to stain sections for 10 min, followed by lead citrate for 10 min and the preparation viewed with a Philips EM 100 transmission electron microscope.

### Statistical Note

All experiments, unless otherwise stated, were performed in triplicate. Where appropriate, a student *t*-test was used to determine the statistical significance of data between the control and different experimental conditions.

## Results

### 3-Hydroxy Fatty Acids Alter the Phagocytic Behavior of Amoebae

In this study, fetuin A was assayed based on prior unpublished work in our group, which revealed that macrophages challenged with 3-hydroxy fatty acids produced this protein. This protein is a major globulin component of serum that has been shown to regulate the function of macrophages (Wang et al., 1998). More to the point, this glycoprotein has been reported to mediate the uptake of particulate material such the uptake of some bacteria (*Escherichia coli* and *Staphylococcus aureus*; both are not known to produce 3-hydroxy fatty acids) including apoptotic cells, by phagocytes (van Oss et al., 1974; Jersmann et al., 2003).

**FIGURE 1:**
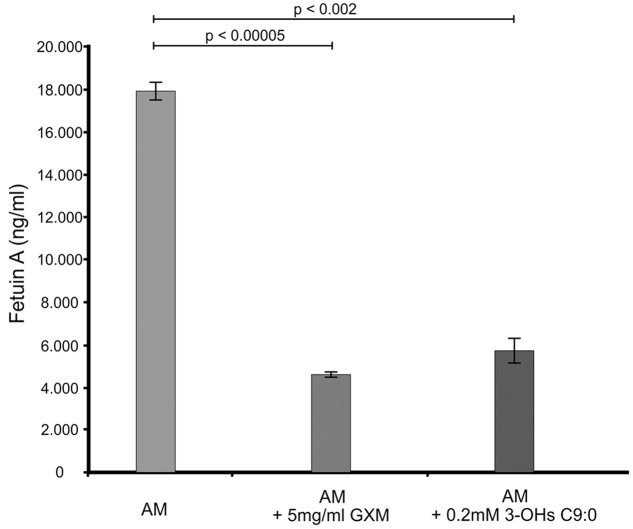
**The ELISA quantitative results showing the levels of fetuin A present across the different experimental conditions i.e., GXM-treated amoeba (AM) and 3-OH C9:0-treated amoeba, including the control i.e., non-treated amoeba cells.** The results indicate that 3-hydroxy C9:0, similar to GXM, may possess an anti-phagocytic quality. GXM, glucuronoxylomannan; 3-OH C9:0, 3-hydroxy C9:0.

The GXM, which was used for comparison purposes in this experiment and others, was successfully isolated (**Supplementary Figure S1**). Our results suggest that amoebae also produce a fetuin A-like protein (**Figure 1**), which may have a similar function as that expressed in macrophages. This assertion is reasonable as macrophages have been theorized to have evolved from amoebae (Siddiqui and Khan, 2012) and in particular, this protein may have evolved from cystatin by gene duplication. Hence, it is conceivable that amoeba may have this protein. When considering the results, it was clear that there was a significant reduction (*p* < 0.01) in the levels of fetuin A when cells were challenged with either GXM (74% reduction) or 3-hydroxy C9:0 (68% reduction) compared to non-treated amoebae. Given the function of this protein, which (among others) is to promote macrophage phagocytosis (Jersmann et al., 2003), the GXM results are conceivable primarily because this molecule is said to be anti-phagocytic (Del Poeta, 2004; Zaragoza et al., 2009). Therefore, by extension, the data suggests that like GXM, 3-hydroxy C9:0 [which is intimately associated with cryptococcal capsules (Sebolai et al., 2007)] may also be anti-phagocytic. This finding may, in part, explain the findings of Madu et al. (2015), wherein they reported that the addition of 3-hydroxy C9:0 to the co-culture media resulted in reduced ability of amoebae to internalize cryptococcal cells compared to the absence of this molecule. Taken together, this points to impairment of the phagocytic process.

### 3-Hydroxy Fatty Acids Protect Cells against Oxidative Damage

**FIGURE 2:**
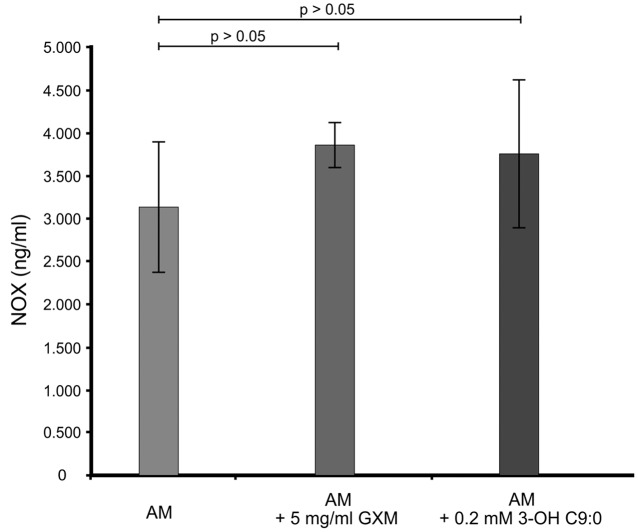
**The ELISA quantitative results showing the levels of NADPH oxidase-1 (NOX) present across the different experimental conditions i.e., GXM-treated amoeba (AM) and 3-OH C9:0-treated amoeba, including the control i.e., non-treated amoeba cells.** There was no significant (*p* > 0.05) difference between the control results and the experimental conditions.

**FIGURE 3:**
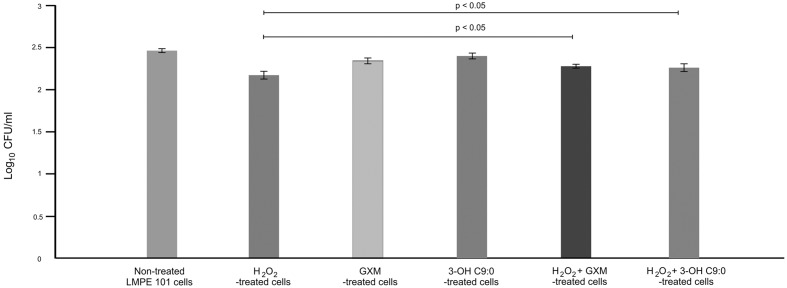
**The survival assay results showing the number of colony forming units (CFUs) that were enumerated after the acapsular strain *C. neoformans* LMPE 101 was exposed to different treatment conditions.** The results suggest that 3-hydroxy C9:0, similar to GXM, protect cells against oxidative damage.

It was first sought to determine if 3-hydroxy C9:0 affected the functioning of NADPH oxidase-1, which could in turn alter the amount of oxidative radicals produced inside the amoebal food vacuoles (**Figure 2**). Here, no meaningful deductions could be made as there was no statistical significance (*p* > 0.05) between the non-treated amoebae and experimental conditions, i.e., 3-hydroxy C9:0-treated amoebae or GXM-treated amoebae. Therefore to compliment this experiment, it was then sought to determine if 3-hydroxy C9:0 could protect cells against the oxidative effects of hydrogen peroxide (when cells were directly challenged) (**Figure 3**). Previously, Zaragoza et al. (2008) showed that GXM protected cells against hydrogen peroxide. Our results confirm this finding. When comparing LMPE 101’s non-treated cells [an acapsular strain that also does not produce 3-hydroxy C9:0 (data not shown)] data to the corresponding hydrogen peroxide-treated cells’, GXM-treated cells’ and 3-hydroxy C9:0-treated cells’ data; significantly fewer CFUs were obtained under the treatment conditions. More importantly, this strain yielded significantly more CFUs (*p* < 0.05) when challenged with hydrogen peroxide in the presence of GXM or 3-hydroxy C9:0 compared to when this strain was challenged with hydrogen peroxide in the absence of the two test molecules. A similar response pattern was observed when examining the results obtained for the strain UOFS Y-1378, which has a capsule (**Supplementary Figure S2**). Also of interest, the levels of hydrogen peroxide in the medium did not dissipate over a 24-h period and remained more or less the same (**Supplementary Figure S3**). This suggests that the reported results specifically for hydrogen peroxide-treated cells and hydrogen peroxide-treated in the presence of GXM or 3-hydroxy C9:0, are not as a result of diminished levels of hydrogen peroxide in the test medium but rather speak to the protective quality of GXM and 3-hydroxy C9:0.

### 3-Hydroxy Fatty Acids Protect Cells against Amoebapore

**FIGURE 4:**
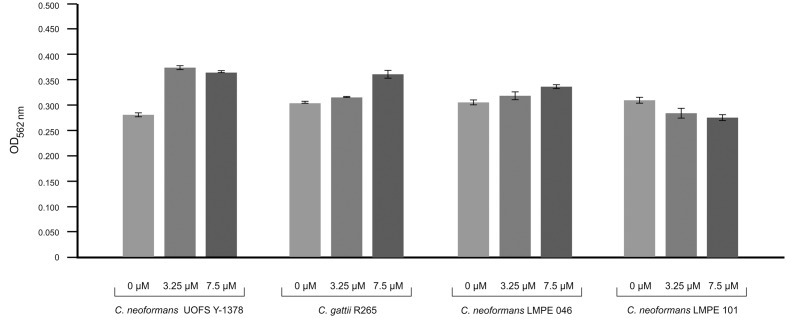
**Results of the effect of amoebapore on the growth of different cryptococcal strains i.e., *C. neoformans* UOFS Y-1378 (has 3-hydroxy C9:0), *C. gattii* R265 (has no 3-hydroxy C9:0), *C. neoformans* LMPE 046 (has 3-hydroxy C9:0), and *C. neoformans* LMPE 101 (is acapsular and has no 3-hydroxy C9:0).** When considering the growth results, it is evident that the strain with 3-hydroxy C9:0 was much more resistant to the effects of amoebapore when compared to the other three strains that do not have 3-hydroxy C9:0.

Amoebapore is an amoeba-specific digest peptide that creates pores in cell walls of targeted cells (Leippe et al., 1994). To test the effectiveness of amoebapore in killing cryptococcal cells, we considered its activity against four strains: (1) one that has a capsule and produces 3-hydroxy C9:0 (UOFS Y-1378), (2) two that have capsules but no 3-hydroxy C9:0 (R265 and LMPE 046), and (3) one without a capsule and 3-hydroxy C9:0 (LMPE 101) (**Figure 4**). Our data show that the strain UOFS Y-1378 was resistant to amoebapore at both test concentrations, and was rather stimulated by this protein. The same phenomenon was observed for the other two capsular strains (R265 and LMPE 046), although the level of growth stimulation was less than for UOFS Y-1378. However, the strain LMPE 101 (which lacks a capsule and 3-hydroxy fatty acids) was sensitive to amoebapore. Based on these results, it is reasonable to conclude that 3-hydroxy C9:0 in UOFS Y-1378 may have acted in concert with the capsule to shield cells hence resulted in the greatest level of resistance that were recorded. On the other hand, the absence of 3-hydroxy fatty acids (in applicable strains) led to a decrease in the levels of resistance. Logically, the strain without 3-hydroxy fatty acids and the capsule was the most susceptible. Nonetheless, an independent study is required to determine if addition of 3-hydroxy fatty acids would lead to strains showing resistance toward amoebapore.

**FIGURE 5:**
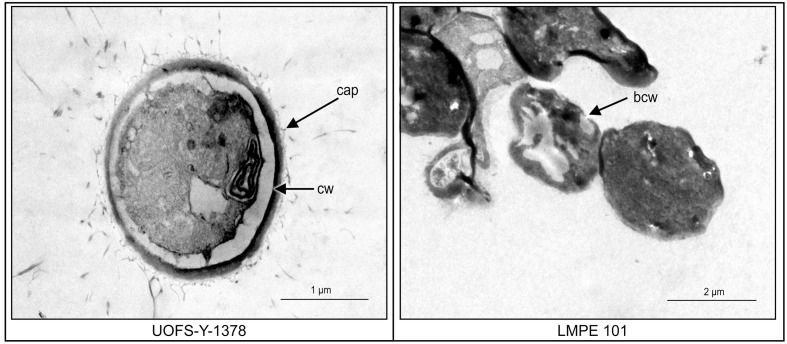
**A comparison of the effect of amoebapore on the ultrastructure of *C. neoformans* UOFS Y-1378 (has 3-hydroxy C9:0) and *C. neoformans* LMPE 101 (is acapsular and has no 3-hydroxy C9:0).** When considering the strain 1378 results, it is conceivable that the expressed resistance may be due to the inability of amoebapore to create pores on its cell wall which is opposite to the results of the strain LMPE 101.

Transmission electron microscopy was subsequently performed on UOFS Y-1378 and LMPE 101 in order to assess the effect of this amoebapore on the ultrastructure of cells. The cell wall of UOFS Y-1378 was without pores while that of LMPE 101 showed a nick in the cell wall (**Figure 5**). The latter could explain the resistance and susceptibility that was, respectively, expressed by UOFS Y-1378 and LMPE 101.

## Discussion

*Cryptococcus neoformans* has evolved to emerge as an important disease-causing microorganism (Casadevall and Perfect, 1998; Levitz and Boekhout, 2006). In part, this is attributed to the ability of cells to subvert the functioning of macrophages. Of concern is how cells are able to take up residency in macrophages, without evoking an immunological response, and subsequently disseminate (Voelz and May, 2010). Some researchers have reasoned that species such amoeba, which predate on microbes like *Cryptococcus*, serve as a pivotal training ground wherein a prey is selected to produce an arsenal of microbial survival factors (Molmeret et al., 2005; Casadevall, 2008).

Traditionally, the capsule has been credited with protecting cells against phagocytic cells (Bose et al., 2003; Del Poeta, 2004; Zaragoza et al., 2009). However, it has emerged that there are other molecules, which may also promote the survival of cryptococcal cells when acted upon phagocytic cells, such as the anti-phagocytic protein-1 (Luberto et al., 2003). Thus, the current study is an extension to the uncovering of other protective molecules, and importantly positions 3-hydroxy fatty acids as being anti-phagocytic in nature. In particular, we showed that these molecules altered the phagocytic behavior of amoebae by affecting fetuin A and protected cells against oxidative damage and amoebapore. These results offer insight into how these molecules may impair the intracellular signaling mechanism, mediated by the fetuin-A-like protein, which is required to initiate internalization. Interactions between fetuin (which is an acute phase protein) and fatty acids are known (Pal et al., 2012). Importantly, the interactions of this protein with mediators that inhibit or promote inflammation or those that inhibit or promote phagocytosis as is the case in the current paper, define its unique properties. Toward this end, it was not surprising to note 3-hydroxy C9:0 decreased the levels of this protein. The effect of 3-hydroxy C9:0 may not be long term and may be abrogated – as it is possible that during an infection, the manifestation of a subclinical inflammation in response to the presence of invading cells may increase its levels. Thus, this may explain how cells may escape phagocytosis (arguably maybe only initially) even though they have been recognized and destined for internalization.

In addition, we have shown how some cells that were unable to escape internalization, could survive the harsh internal environment (hydrogen peroxide) found inside the food vacuole of amoebae or phagosomes of macrophages. The role of lipids in preventing oxidative damage is not clear cut. Typically, a radical like hydrogen peroxide (has a predilection of targeting double bonds of unsaturated fatty acids) can enter a cell by simple diffusion and gain access to its membranes. Based on the results we recorded in the current study, it is possible that 3-hydroxy C9:0 may have been incorporated into the membranes and in turn, influenced the fluidity. In his dissertation, Mochochoko showed that treatment of *Pseudomonas* cells with 3-hydroxy C9:0 impaired membrane fluidity as cells were unable to traffic molecules in and out (unpublished data). In such a scenario, hydrogen peroxide (which can easily diffuse into a cell) may have been prevented from entering the cells hence its ineffectiveness to reduce CFU counts in the presence of 3-hydroxy C9:0.

There are also lipid-soluble non-enzymatic antioxidants such as tocopherol, which can neutralize the effect of hydrogen peroxide. To the point, this molecule can be targeted and oxidized by reactive oxygen species like hydrogen peroxide, and in turn prevent lipid peroxidation (Latifi et al., 2009). Based on the latter, it is also possible that after incorporation of 3-hydroxy C9:0 into membranes, these molecules may like-wise be targeted and oxidized – hence the effect of hydrogen peroxide was abrogated. With respect to observed amoebapore results, it is possible that 3-hydroxy C9:0 may form a complex with this antimicrobial peptide to neutralize it. Lipids have previously been shown to block the activity of many antimicrobial peptides that exhibit membrane disrupting properties (Malanovic and Lohner, 2016). In their paper, these authors further make the point that trapping of antimicrobial peptides sufficiently reduces the total concentration of these peptides on the cytoplasmic membrane.

Taken together, these findings may contribute to our understanding of how cryptococcal cells may survive amoebal phagocytosis as well as provide insight into survival mechanisms inside phagosomes of macrophages during dissemination. This is supported by the idea that cryptococcal cell would recognize both amoebae and macrophages as the same predatory cell (Steenbergen et al., 2001; Steenbergen and Casadevall, 2003), and thus this fungus will accordingly display the same defensive behavior in order to escape phagocytic processing.

## Conclusion

The presented results highlight the importance of 3-hydroxy fatty acids to the pathogenesis of *C. neoformans*. It will be interesting to see if similar results could be observed in macrophages as well as in laboratory animals, and additionally, if creation of a 3-hydroxy fatty acid-deficient mutant [via deletion of beta-oxidation gene(s)] will result in a less virulent strain that is susceptible to amoeba or macrophage action.

## Author Contributions

All authors contributed significantly to the paper and all authors are in agreement with the content of the manuscript. UM performed the experiments. AO, CP, and JA provided strategic inputs. OS designed and provided facilities and resources to complete the study. UM, AO, CP, JA, and OS wrote the manuscript.

## Conflict of Interest Statement

The authors declare that the research was conducted in the absence of any commercial or financial relationships that could be construed as a potential conflict of interest.

## Supplementary Material

**FIGURE S1 | (A)** Shows a positive agglutination test for the isolated GXM while **(B)** also shows a positive ELISA result for the isolated GXM. Both these tests confirm the successful isolation of GXM in our study after it was harvested using irradiation.

**FIGURE S2 | The survival assay results showing the number of colony forming units (CFUs) that were enumerated after the strain *C. neoformans* UOFS Y-1378 was exposed to different treatment conditions.** The results suggest that 3-hydroxy C9:0, similar to GXM, protect cells against oxidative damage.

**FIGURE S3 | The fate of hydrogen peroxide in media containing hydrogen peroxide-treated cells as well as hydrogen peroxide-treated cells in the presence of GXM or 3-hydroxy C9:0.** **(A)** Shows results for the strain *C. neoformans* LMPE 101 while **(B)** shows results for the strain *C. neoformans* UOFS Y-1378.
