# Supplementary figures and images for: Elucidation of the Role of 3-Hydroxy Fatty Acids in Cryptococcus-amoeba Interactions
Source: Front Microbiol. 2017 Apr 26;8:765. doi: 10.3389/fmicb.2017.00765 (PMC5405085; doi:10.3389/fmicb.2017.00765)

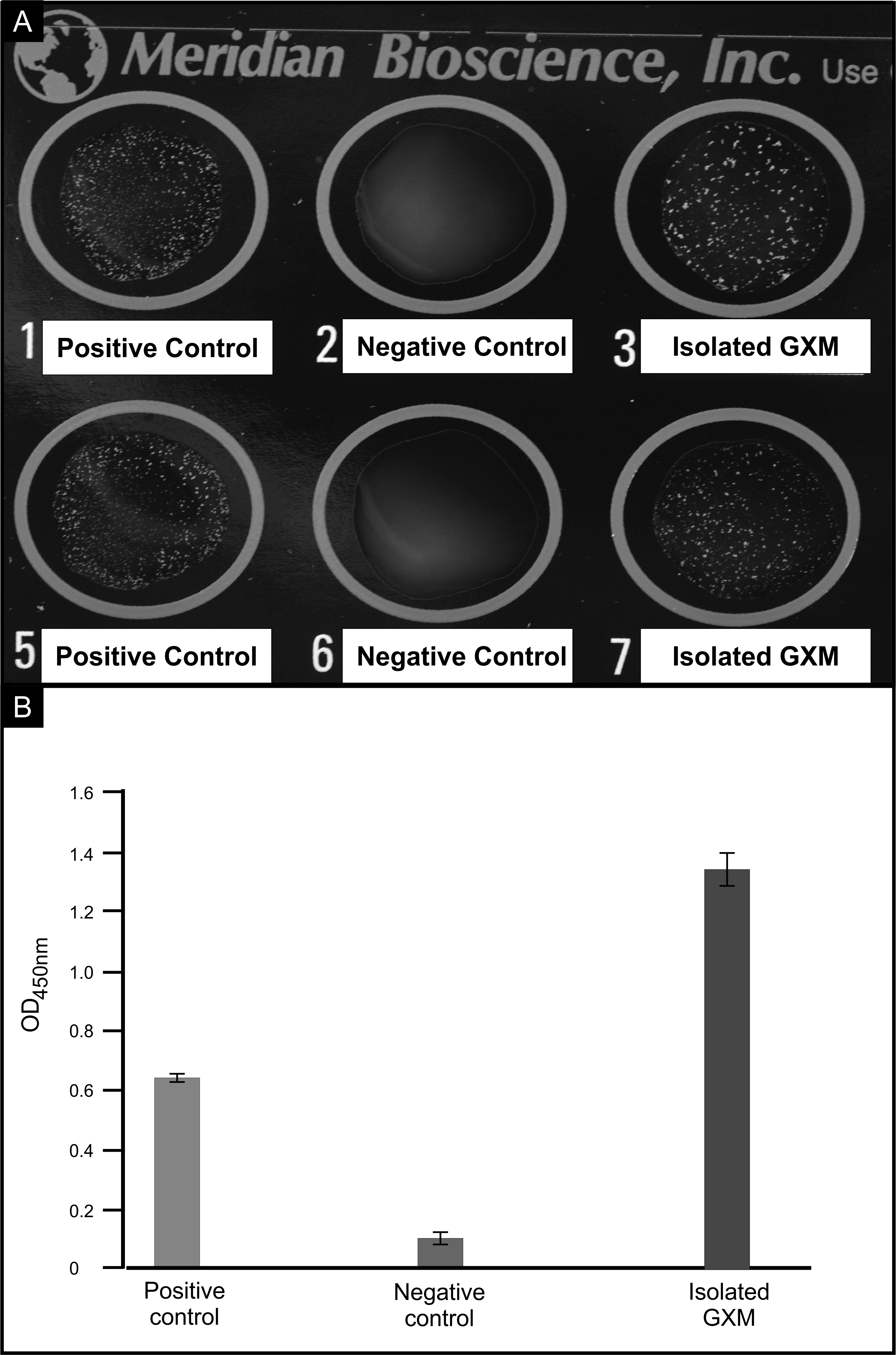

Supplement: Supplementary file 1 [file Image_1.JPEG]

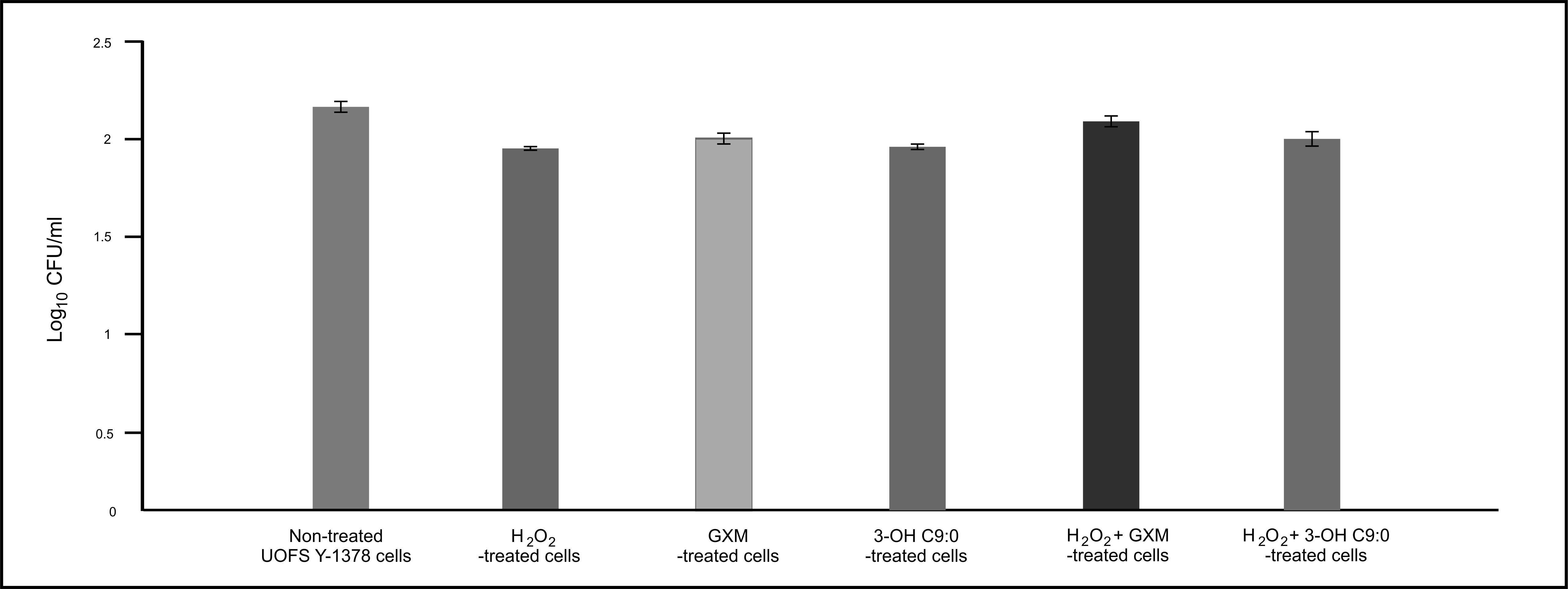

Supplement: Supplementary file 2 [file Image_2.JPEG]

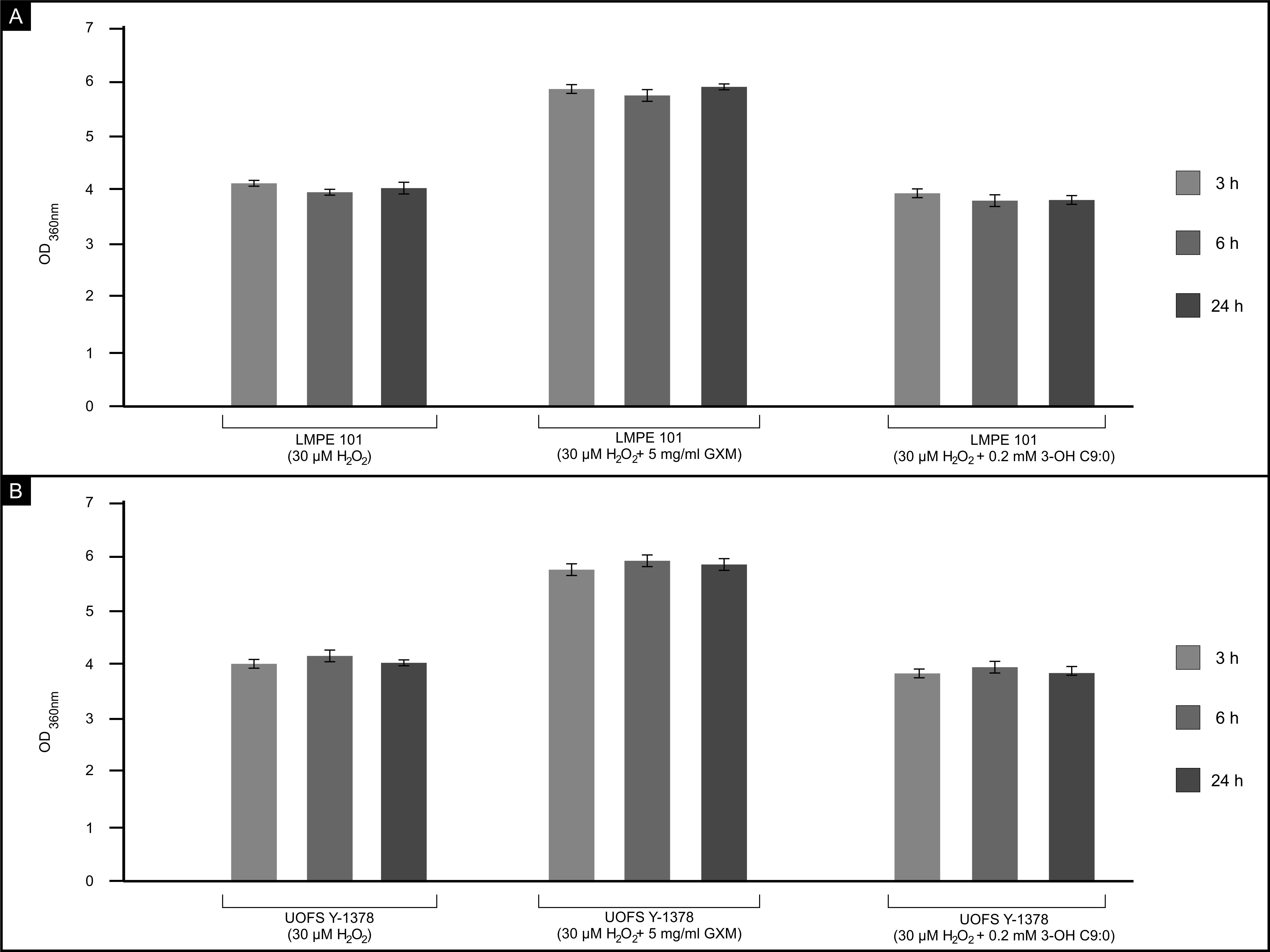

Supplement: Supplementary file 3 [file Image_3.JPEG]
